# Network pharmacology approach and experimental verification of Dan-Shen Decoction in the treatment of ischemic heart disease

**DOI:** 10.1080/13880209.2022.2152059

**Published:** 2022-12-22

**Authors:** Difei Gong, Tianyi Yuan, Ranran Wang, Shuchan Sun, Awaguli Dawuti, Shoubao Wang, Guanhua Du, Lianhua Fang

**Affiliations:** State Key Laboratory of Bioactive Substances and Functions of Natural Medicines, Institute of Materia Medica, Chinese Academy of Medical Sciences and Peking Union Medical College, Beijing, China

**Keywords:** Molecular docking, H9c2 cells, mechanism

## Abstract

**Context:**

Dan-Shen Decoction, which is composed of Danshen, Tanxiang and Sharen, has a good therapeutic effect on ischemic heart disease (IHD). However, systematic research on the exact mechanism of action of Dan-Shen Decoction is still lacking. The anti-IHD effect of Dan-Shen Decoction was examined in this study using a systematic pharmacological method.

**Objective:**

This study validates the efficacy and explores the potential mechanisms of Dan-Shen Decoction in treating IHD by integrating network pharmacology analyses and experimental verification.

**Materials and methods:**

The active components, critical targets and potential mechanisms of Dan-Shen Decoction against IHD were predicted by network pharmacology and molecule docking. H9c2 cells were pretreated with various 1 µg/mL Dan-Shen Decoction for 2 h before induction with 1000 µmol/L CoCl_2_ for 24 h. The cell viability was detected by CCK8, and protein expression was detected by western blots.

**Results:**

The network pharmacology approach successfully identified 69 active components in Dan-Shen Decoction, and 122 potential targets involved in the treatment of IHD. The *in vitro* experiments indicate that the anti-IHD effect of Dan-Shen Decoction may be closely associated with targets such as AKT1 and MAPK1, as well as biological processes such as cell proliferation, inflammatory response, and metabolism.

**Conclusions:**

This study not only provides new insights into the mechanism of Dan-Shen Decoction against IHD, but also provides important information and new research ideas for the discovery of anti-IHD compounds from traditional Chinese medicine.

## Introduction

Cardiovascular diseases are major life-threatening diseases, and the death rate from ischemic heart disease (IHD) is increasing. In 2019, the number of deaths caused by IHD worldwide reached 9.14 million, which is expected to rise in the future (Roth et al. 2020). Therefore, there is an urgent need to prevent and treat IHD. IHD is characterized by an imbalance in myocardial blood supply and demand, which results in the death of cardiac cells. Heart attack, or myocardial infarction (MI), is the most common manifestation of IHD that results in cardiac cell death (Hausenloy and Yellon 2016).

Currently, some drug classes, such as β-receptor blockers, calcium channel blockers, diuretics, nitrates and aspirin, are commonly used in the treatment of IHD. Percutaneous coronary intervention (PCI) and coronary artery bypass grafting (CABG) decrease ischemia to a greater extent than medical therapy alone (Boden et al. 2007). However, clinical outcomes have not improved commensurately. As a result, there is an urgent need to develop complementary and alternative therapies for major heart diseases (Pratt 2010). For thousands of years, traditional Chinese medicine, particularly herbal compounding, has been the most commonly used treatment for cardiovascular disease in China (Liu et al. 2011). As patients with IHD have different clinical features or symptoms, different Chinese herbal formulas are used for treatment.

Dan-Shen Decoction is made up of three Chinese herbal medicines, namely *Salvia miltiorrhiza* Bunge (Lamiaceae) (Danshen), *Santalum album* L. (Santalaceae) (Tanxiang) and *Amomum villosum* Lour. (Zingiberaceae) (Sharen), all of which have been shown to activate blood circulation and eliminate blood stasis (Kong and Du 2019). The plant name has been checked with MPNS (http://mpns.kew.org). Dan-Shen Decoction was first mentioned in the book of Shi Fang Ge Kuo for the first time in 1801 A.D., with the comment, "Dan-Shen Decoction has multiple effects on heartache and epigastric pain, especially for women". A large body of evidence suggests that Dan-Shen Decoction appears to have a wide range of pharmacological effects on the cardiovascular system. Pretreatment with Dan-Shen Decoction significantly lowers serum myocardial enzyme levels, reduces myocardial infarct area, lowers inflammatory factor expression and protects cardiomyocytes from ischemia/reperfusion injury (Hoo et al. 2021; Ren et al. 2021). The specific mechanism of Dan-Shen Decoction could be used to treat myocardial ischemia by inhibiting the NLR family pyrin domain containing 3 (NLRP3) inflammatory vesicles, affecting the expression of apoptosis-related genes and regulating nitric oxide and endothelin levels (Wang and Li 2009; Luo et al. 2011; Yan et al. 2021).

Considering the studies mentioned above, Dan-Shen Decoction appears to play a significant preventive and therapeutic role in IHD. Mechanistically, the effects of Dan-Shen Decoction are associated with multiple targets and signalling pathways. This study investigates possible mechanisms using network pharmacology and validates them using cell experiments to elucidate the effects of Dan-Shen Decoction in IHD treatment.

## Materials and methods

### Network pharmacology

#### Targets collection of Dan-Shen Decoction

Potential targets of Dan-Shen Decoction were identified using the Traditional Chinese Medicine Systematic Pharmacology Database and Analysis Platform (TCMSP), the Integrative Pharmacology-based Research Platform of Traditional Chinese Medicine (TCMIP), and the Bioinformatics Analysis Tool for Molecular mechanism of Traditional Chinese Medicine (BATMAN-TCM) and then normalized by the UniProt database (Ru et al. 2014; Liu et al. 2016; Xu et al. 2019).

#### Identification of IHD-related targets

The GeneCards database (http://www.genecards.org), the GenCLiP 2.0 webserver (http://ci.smu.edu.cn), and the Comparative Toxicogenomics Database (http://ctdbase.org/) were accessed to collect IHD-related genes using the search terms ‘ischemic heart disease’ and the bioinformatics platform (http://www. bioinformatics.com.cn) was used to screen the intersection of genes between the active ingredient targets and disease targets (Huang et al. 2008; Wang et al. 2014; Davis et al. 2021; Safran et al. 2021).

#### Construction of an active ingredient-target-disease network

Cytoscape 3.7.1 software was used to construct a network of the active components and the key targets, in which nodes represent active components, targets, diseases and drug names, while edges represent interactions between nodes. The network elucidates the interaction between the active components of Dan-Shen Decoction and the targets against IHD.

#### Construction and analysis of the PPI network

The intersection of drug targets and IHD-related targets was imported into the String platform (https://www.string-db.org) for protein–protein interaction analysis (Szklarczyk et al. 2019). The protein–protein interaction network (PPI network) was constructed and the top 30 potential targets of Dan-Shen Decoction against IHD were identified.

#### GO and KEGG enrichment analysis

The Metascape database (http://metascape.org/) was used to conduct Gene Ontology (GO) enrichment and Kyoto Encyclopedia of Genes and Genomes (KEGG) pathway analyses for the potential therapeutic targets of Dan-Shen Decoction against IHD (Zhou et al. 2019). KEGG pathway analyses were performed to obtain the signalling pathways, which further explained the mechanism of the anti-IHD effect.

### Molecular docking

The TCMSP platform provided mol2 format files of luteolin, przewaquinone E, dihydrotanshinlactone, isorhamnetin and β-sitosterol, while the protein data bank (PDB) (http://www.rcsb.org) provided PDB format files of AKT1 (PDB ID: 3O96) and MAPK1 (PDB ID: 6G54). Molecular docking was performed using DS BIOVIA Discovery Studio Client 2018 v18.1 software. Water molecules were removed and loops were added after the protein structure was prepared. The CDOCKER method was then used to calculate the interaction energy and binding mode.

### Experimental verification

#### Drug preparation and reagents

Homemade Dan-Shen Decoction granules were prepared according to a previous study (Yan et al. 2021). We used DMSO to dissolve and dilute with normal saline to prepare a 20 mg/mL Dan-Shen Decoction storage solution, which was stored at −20 °C.

PBS (Leagene, 0804A21); DMEM (Gibco, 8120365); fetal bovine serum FBS (Procell, SA210518); EDTA trypsin (Beyotime, C0203-100 mL); penicillin–streptomycin mixed solution PS (Solarbio, 20200617); CCK-8 (Dojindo, PN534); CoCl_2_ (Sigma, SLCF6741); Antibody c-JUN (9165), SAPK/JNK (9252), PI3 Kinase p110α (4249), PI3 Kinase p85 (4257), NF-κB (8242), p-NF-κB p65 (3033), all from Cell Signalling Technology, dilution ratio: 1:1000; β-actin (Proteintech, 60008-1-IG, dilution ratio: 1: 2000); STAT3 (Santa Cruz Biotechnology, 8019, dilution ratio: 1:100); p-c-JUN (Santa Cruz Biotechnology, sc-822, dilution ratio: 1:100); Goat Anti Rabbit IgG-HRP (Gene–Protein Link, P03S02); Goat Anti mouse IgG-HRP (Gene–Protein Link, P03S01).

#### Cell culturing

The rat H9c2 cardiomyocyte cell line was purchased from the American Type Culture Collection (ATCC). H9c2s were cultured in Dulbecco’s modified Eagle’s medium (DMEM) (Gibco, Aukland, New Zealand) supplemented with 10% fetal bovine serum (FBS) and 1% penicillin and streptomycin (PS). The cells were cultured at 37 °C with 5% CO_2_ in humidified conditions. All experiments were carried out with cells at passages 4–8.

#### Cell viability assay

H9c2 cardiomyocytes were seeded at a cell density of 8000 cells/well in 96-well plates and incubated overnight. Then, different concentrations of Dan-Shen Decoction and CoCl_2_ were added for 24 h. Subsequently, CCK-8 solution was added to each well, after incubation at 37 °C for 90 min. The optical density OD450 was measured by a microplate reader.

#### Western blot

H9c2 cardiomyocytes were lysed in precooled RIPA buffer containing phosphatase inhibitor cocktail (1:100), protein phosphatase inhibitor (1:50) and PMSF (1:200) on ice for 30 min. After collecting the supernatant, the protein concentration was determined using the BCA Protein Assay Kit. Protein samples were loaded onto a 10% SDS-polyacrylamide gel and transferred to the PVDF membrane. After blocking with TBST containing 5% bovine serum albumin (BSA) for 2 h, the membrane was incubated with specific primary antibodies overnight at 4 °C. The membrane was washed five times with TBST and incubated with the corresponding HRP-conjugated secondary antibodies (1:5000) for 2 h at room temperature. After washing with TBST, the intensity of the scanned bands was measured with enhanced chemiluminescence detection reagents and analyzed by Image Lab software. Targeted bands were normalized to β-actin.

#### Mathematical and statistical analyses

Statistical analyses were performed using GraphPad 9.1.1. The data are reported as the mean ± SEM. Dunnett’s multiple comparisons test in one-way ANOVA was used for multiple comparisons between groups, and a *p* value < 0.05 was considered statistically significant.

## Results

### Network pharmacology

#### Active components and potential targets

**Figure 1.**
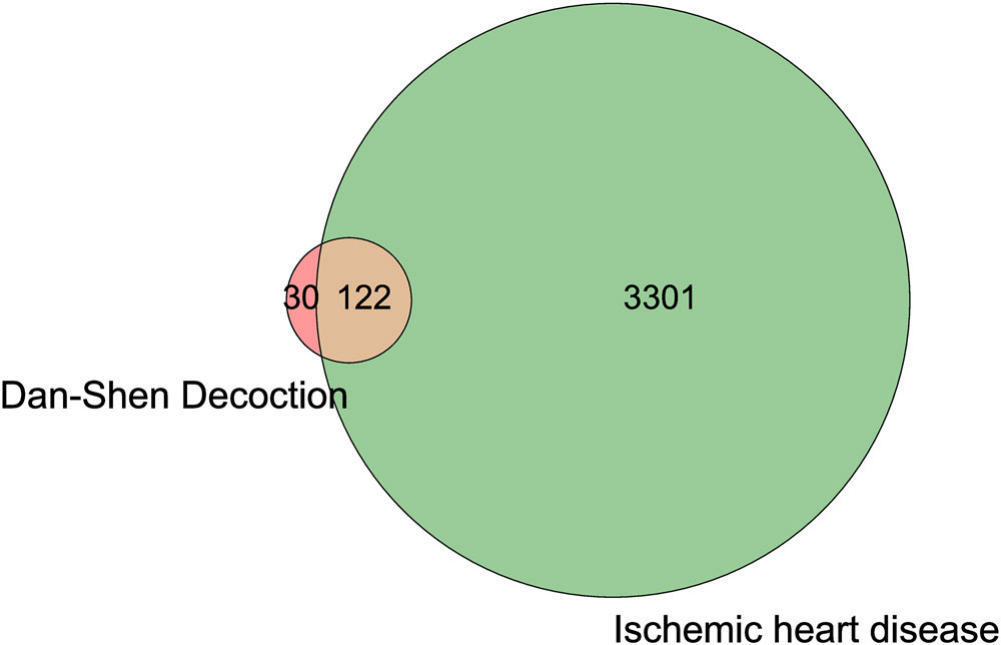
Venn diagram of the targets of Dan-Shen Decoction and IHD.

A total of 58, 3 and 8 active components in Danshen, Tanxiang, and Sharen were obtained through the TCMSP, TCMIP, and BATMAN-TCM databases and screened by OB ≥ 30% and DL ≥ 0.18, respectively (Table 1). Their corresponding target genes were 152 in total, and the GeneCards database was used to obtain 3423 ischemic heart disease targets. A total of 122 intersecting targets were obtained by combining the targets of Dan-Shen Decoction and IHD (Figure 1) (Bardou et al. 2014).

**Table 1.** Main active components of Dan-Shen Decoction.

Drug	Name	MOL ID	Active components	OB(100 %)	DL
Danshen	DS1	MOL001601	1,2,5,6-Tetrahydrotanshinone	38.75	0.36
Danshen	DS2	MOL001659	Poriferasterol	43.83	0.76
Danshen	DS3	MOL001771	Poriferast-5-en-3beta-ol	36.91	0.75
Danshen	DS4	MOL001942	Isoimperatorin	45.46	0.23
Danshen	DS5	MOL002222	Sugiol	36.11	0.28
Danshen	DS6	MOL002651	Dehydrotanshinone II A	43.76	0.4
Danshen	DS7	MOL002776	Baicalin	40.12	0.75
Danshen	DS8	MOL000569	Digallate	61.85	0.26
Danshen	DS9	MOL000006	Luteolin	36.16	0.25
Danshen	DS10	MOL007036	5,6-Dihydroxy-7-isopropyl-1,1-dimethyl-2,3-dihydrophenanthren-4-one	33.77	0.29
Danshen	DS11	MOL007041	2-Isopropyl-8-methylphenanthrene-3,4-dione	40.86	0.23
Danshen	DS12	MOL007045	3α-HydroxytanshinoneIIa	44.93	0.44
Danshen	DS13	MOL007048	(*E*)-3-[2-(3,4-Dihydroxyphenyl)-7-hydroxy-benzofuran-4-yl]acrylic acid	48.24	0.31
Danshen	DS14	MOL007049	4-Methylenemiltirone	34.35	0.23
Danshen	DS15	MOL007050	2-(4-Hydroxy-3-methoxyphenyl)-5-(3-hydroxypropyl)-7-methoxy-3-benzofurancarboxaldehyde	62.78	0.4
Danshen	DS16	MOL007058	Formyltanshinone	73.44	0.42
Danshen	DS17	MOL007059	3-beta-Hydroxymethyllenetanshiquinone	32.16	0.41
Danshen	DS18	MOL007061	Methylenetanshinquinone	37.07	0.36
Danshen	DS19	MOL007063	Przewalskin a	37.11	0.65
Danshen	DS20	MOL007064	Przewalskin b	110.32	0.44
Danshen	DS21	MOL007068	Przewaquinone B	62.24	0.41
Danshen	DS22	MOL007069	Przewaquinone c	55.74	0.4
Danshen	DS23	MOL007070	(6*S*,7*R*)-6,7-Dihydroxy-1,6-dimethyl-8,9-dihydro-7H-naphtho[8,7-*g*]benzofuran-10,11-dione	41.31	0.45
Danshen	DS24	MOL007071	Przewaquinone f	40.31	0.46
Danshen	DS25	MOL007077	Sclareol	43.67	0.21
Danshen	DS26	MOL007079	Tanshinaldehyde	52.47	0.45
Danshen	DS27	MOL007081	Danshenol B	57.95	0.56
Danshen	DS28	MOL007082	Danshenol A	56.97	0.52
Danshen	DS29	MOL007085	Salvilenone	30.38	0.38
Danshen	DS30	MOL007088	Cryptotanshinone	52.34	0.4
Danshen	DS31	MOL007093	Dan-shexinkum d	38.88	0.55
Danshen	DS32	MOL007094	Danshenspiroketallactone	50.43	0.31
Danshen	DS33	MOL007098	Deoxyneocryptotanshinone	49.4	0.29
Danshen	DS34	MOL007100	Dihydrotanshinlactone	38.68	0.32
Danshen	DS35	MOL007101	DihydrotanshinoneI	45.04	0.36
Danshen	DS36	MOL007105	*epi*-Danshenspiroketallactone	68.27	0.31
Danshen	DS37	MOL007107	C09092	36.07	0.25
Danshen	DS38	MOL007108	Isocryptotanshi-none	54.98	0.39
Danshen	DS39	MOL007111	Isotanshinone II	49.92	0.4
Danshen	DS40	MOL007119	Miltionone I	49.68	0.32
Danshen	DS41	MOL007120	Miltionone II	71.03	0.44
Danshen	DS42	MOL007121	Miltipolone	36.56	0.37
Danshen	DS43	MOL007122	Miltirone	38.76	0.25
Danshen	DS44	MOL007124	Neocryptotanshinone ii	39.46	0.23
Danshen	DS45	MOL007125	Neocryptotanshinone	52.49	0.32
Danshen	DS46	MOL007127	1-Methyl-8,9-dihydro-7H-naphtho[5,6-*g*]benzofuran-6,10,11-trione	34.72	0.37
Danshen	DS47	MOL007130	Prolithospermic acid	64.37	0.31
Danshen	DS48	MOL007132	(2*R*)-3-(3,4-Dihydroxyphenyl)-2-[(*Z*)-3-(3,4-dihydroxyphenyl)acryloyl]oxy-propionic acid	109.38	0.35
Danshen	DS49	MOL007141	Sal*via*nolic acid g	45.56	0.61
Danshen	DS50	MOL007142	Sal*via*nolic acid j	43.38	0.72
Danshen	DS51	MOL007143	Salvilenone I	32.43	0.23
Danshen	DS52	MOL007145	Salviolone	31.72	0.24
Danshen	DS53	MOL007150	(6*S*)-6-Hydroxy-1-methyl-6-methylol-8,9-dihydro-7H-naphtho[8,7-*g*]benzofuran-10,11-quinone	75.39	0.46
Danshen	DS54	MOL007151	Tanshindiol B	42.67	0.45
Danshen	DS55	MOL007152	Przewaquinone E	42.85	0.45
Danshen	DS56	MOL007154	Tanshinone iia	49.89	0.4
Danshen	DS57	MOL007155	(6*S*)-6-(Hydroxymethyl)-1,6-dimethyl-8,9-dihydro-7H-naphtho[8,7-*g*]benzofuran-10,11-dione	65.26	0.45
Danshen	DS58	MOL007156	Tanshinone VI	45.64	0.3
Tanxiang	TX1	MOL000354	Isorhamnetin	49.6	0.31
Tanxiang	TX2	MOL000006	Luteolin	36.16	0.25
Tanxiang	TX3	MOL002322	Isovitexin	31.29	0.72
Sharen	SR1	MOL001755	24-Ethylcholest-4-en-3-one	36.08	0.76
Sharen	SR2	MOL001771	Poriferast-5-en-3beta-ol	36.91	0.75
Sharen	SR3	MOL001973	Sitosteryl acetate	40.39	0.85
Sharen	SR4	MOL000358	beta-Sitosterol	36.91	0.75
Sharen	SR5	MOL000449	Stigmasterol	43.83	0.76
Sharen	SR6	MOL007180	Vitamin e	32.29	0.7
Sharen	SR7	MOL007535	(5*S*,8*S*,9*S*,10*R*,13*R*,14*S*,17*R*)-17-[(1*R*,4*R*)-4-Ethyl-1,5-dimethylhexyl]-10,13-dimethyl-2,4,5,7,8,9,11,12,14,15,16,17-dodecahydro-1H-cyclopenta[*a*]phenanthrene-3,6-dione	33.12	0.79
Sharen	SR8	MOL007536	Stigmasta-5,22-dien-3-beta-yl acetate	46.44	0.86

#### Active component-target-disease network

**Figure 2.**
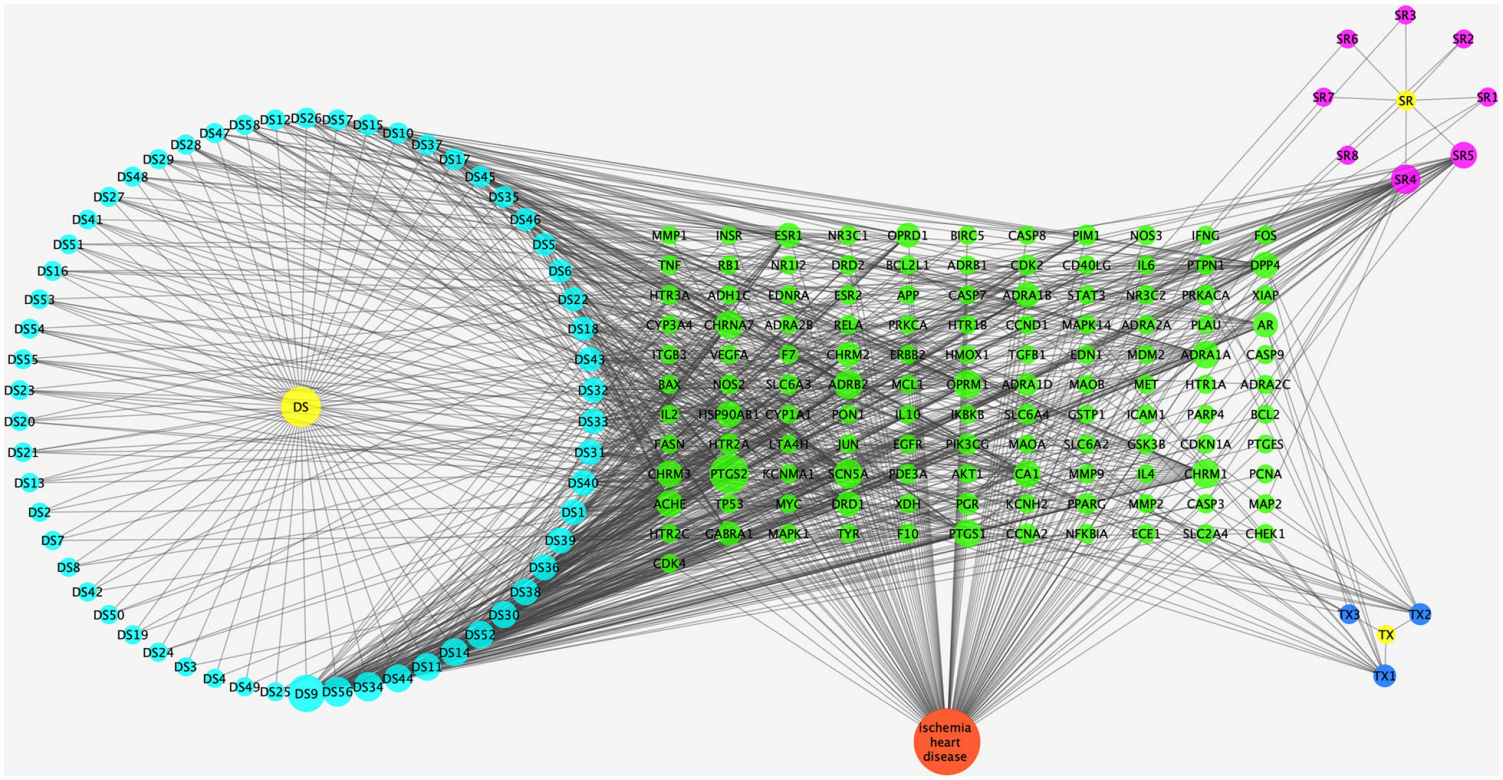
Relationship network between potential therapeutic targets against IHD and active components of Dan-Shen Decoction (DS: Danshen; TX: Tanxiang; SR: Sharen).

A relationship network was built between potential therapeutic targets against IHD and active components of Dan-Shen Decoction (Figure 2). We used the NetworkAnalyzer built into Cytoscape 3.7.1 software to analyze the node degree values. Among them, the active components with a high number of connected targets in Danshen were luteolin (degree = 51), przewaquinone E (degree = 33) and dihydrotanshinlactone (degree = 32), and the active components with more targets in Tanxiang and Sharen were isorhamnetin (degree = 16) and β-sitosterol (degree = 32). In addition, the targets connecting more active components included prostaglandin-endoperoxide synthase 2 (PTGS2) (degree = 56), sodium voltage-gated channel alpha subunit 5 (SCN5A) (degree = 32), adrenoceptor beta 2 (ADRB2) (degree = 32), μ-opioid receptor mu 1 (OPRM1) (degree = 30) and cholinergic receptor muscarinic 1 (CHRM1) (degree = 30).

#### Protein–protein interaction network

**Figure 3.**
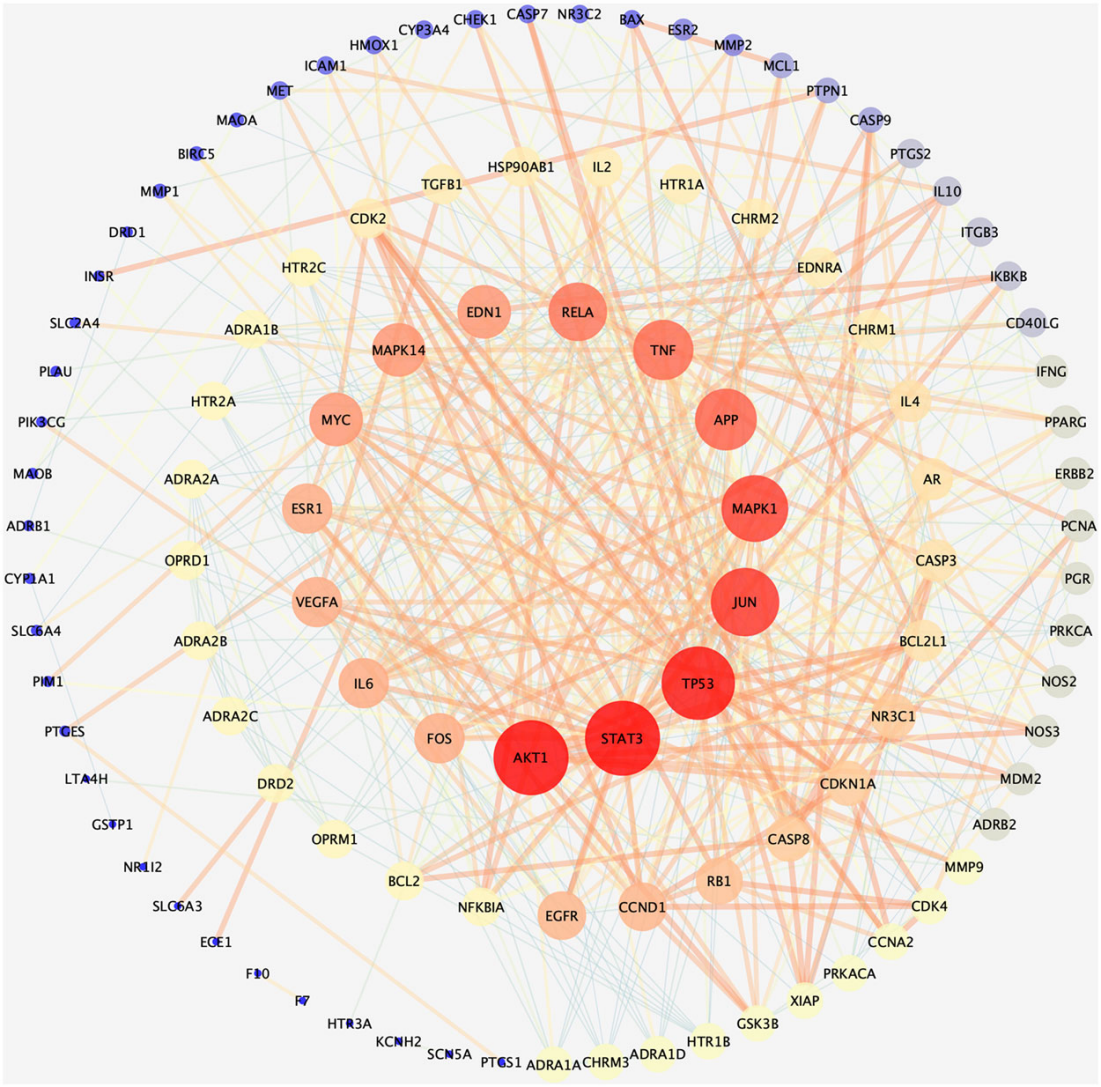
PPI network of potential targets of Dan-Shen Decoction against IHD (inner circle is the top 15 hub proteins in degree value).

The PPI network of potential targets of Dan-Shen Decoction against IHD was constructed using the String platform (Figure 3), and the top 15 proteins in degree value are shown in Table 2. Mitogen-activated protein kinase 1/14 (MAPK1/14), endothelin 1 (EDN1), interleukin-6 (IL-6), vascular endothelial growth factor A (VEGFA) and epidermal growth factor receptor (EGFR) were discovered to be very important in the network by analyzing network topology parameters.

**Table 2.** Potential targets of Dan-Shen Decoction for the treatment of IHD.

Gene	Target	Degree
AKT1	RAC-alpha serine/threonine-protein kinase	32
STAT3	Signal transducer and activator of transcription 3	32
TP53	Cellular tumour antigen p53	31
JUN	Transcription factor AP-1	28
MAPK1	Mitogen-activated protein kinase 1	27
APP	Amyloid beta A4 protein	24
TNF	Tumour necrosis factor	23
RELA	Transcription factor p65	22
EDN1	Endothelin-1	19
MAPK14	Mitogen-activated protein kinase 14	19
MYC	Myc proto-oncogene protein	19
ESR1	Estrogen receptor	17
VEGFA	Vascular endothelial growth factor A	17
IL6	Interleukin-6	17
FOS	Proto-oncogene c-Fos	17

#### Functional modules

**Figure 4.**
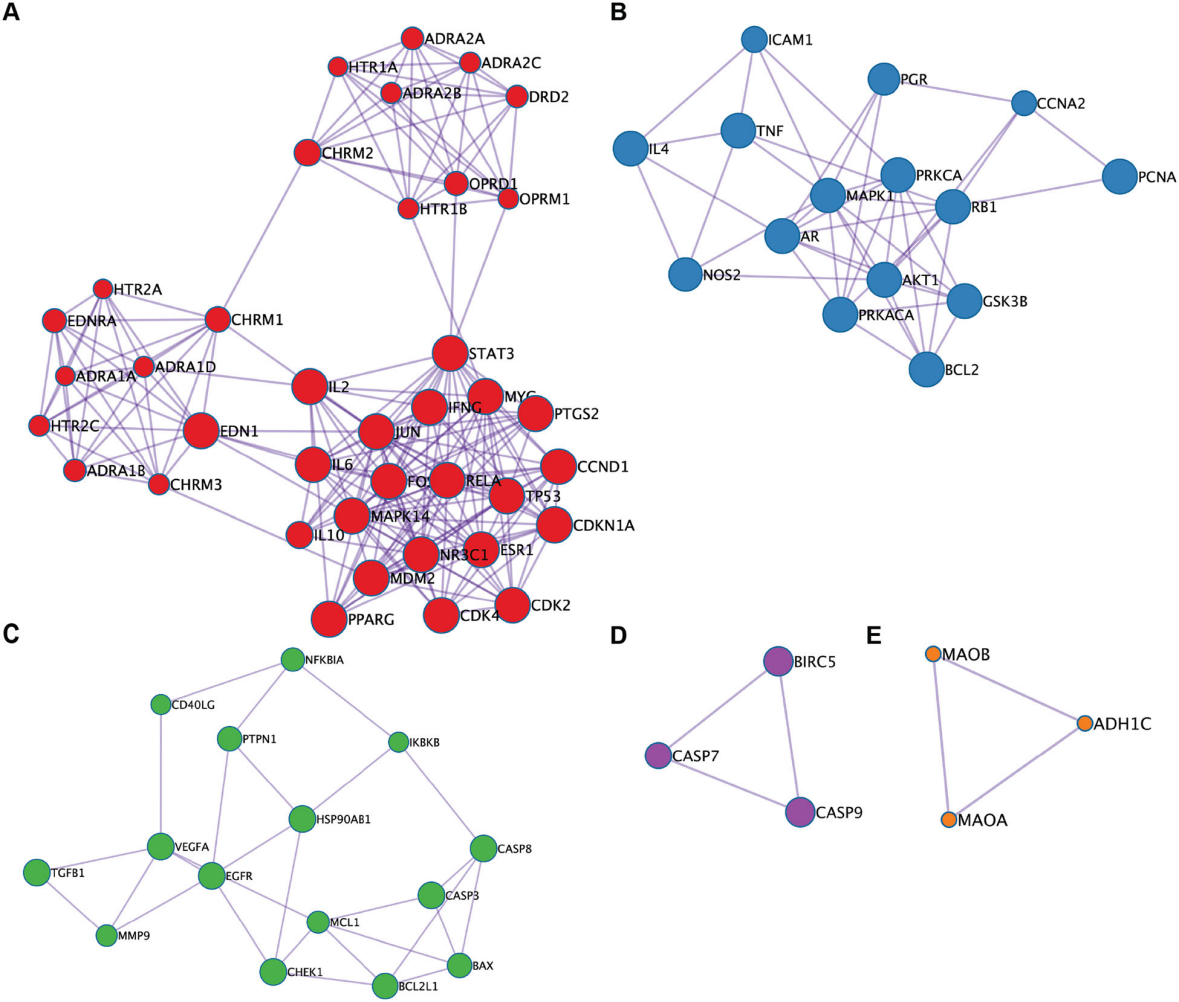
Key functional modules of Dan-Shen Decoction against IHD.

Five biological functional modules were identified when the PPI network was analyzed using the MCODE algorithm (Figure 4). GO and KEGG pathway enrichment analyses were performed on the obtained modules (Table 3), the findings of which included the ‘G protein-coupled receptor signalling pathway’, ‘apoptosis signalling pathway’, ‘regulatory processes of cellular stress’ and ‘metabolic processes of organic hydroxyl compounds’.

**Table 3.** GO and KEGG enrichment analyses of functional modules.

Sub-network	Annotation
MCODE A	GO:0007187, G protein-coupled receptor signalling pathway, coupled to cyclic nucleotide second messenger
	GO:0007188, adenylate cyclase-modulating G protein-coupled receptor signalling pathway
	GO:0007200, phospholipase C-activating G protein-coupled receptor signalling pathway
MCODE B	GO:2001237, negative regulation of extrinsic apoptotic signalling pathway
	GO:2001234, negative regulation of apoptotic signalling pathway
	GO:2001236, regulation of extrinsic apoptotic signalling pathway
MCODE C	GO:0080135, regulation of cellular response to stress
	GO:0097190, apoptotic signalling pathway
	GO:0097191, extrinsic apoptotic signalling pathway
MCODE E	GO:1901615, organic hydroxy compound metabolic process

#### GO enrichment and KEGG pathway analyses

**Figure 5.**
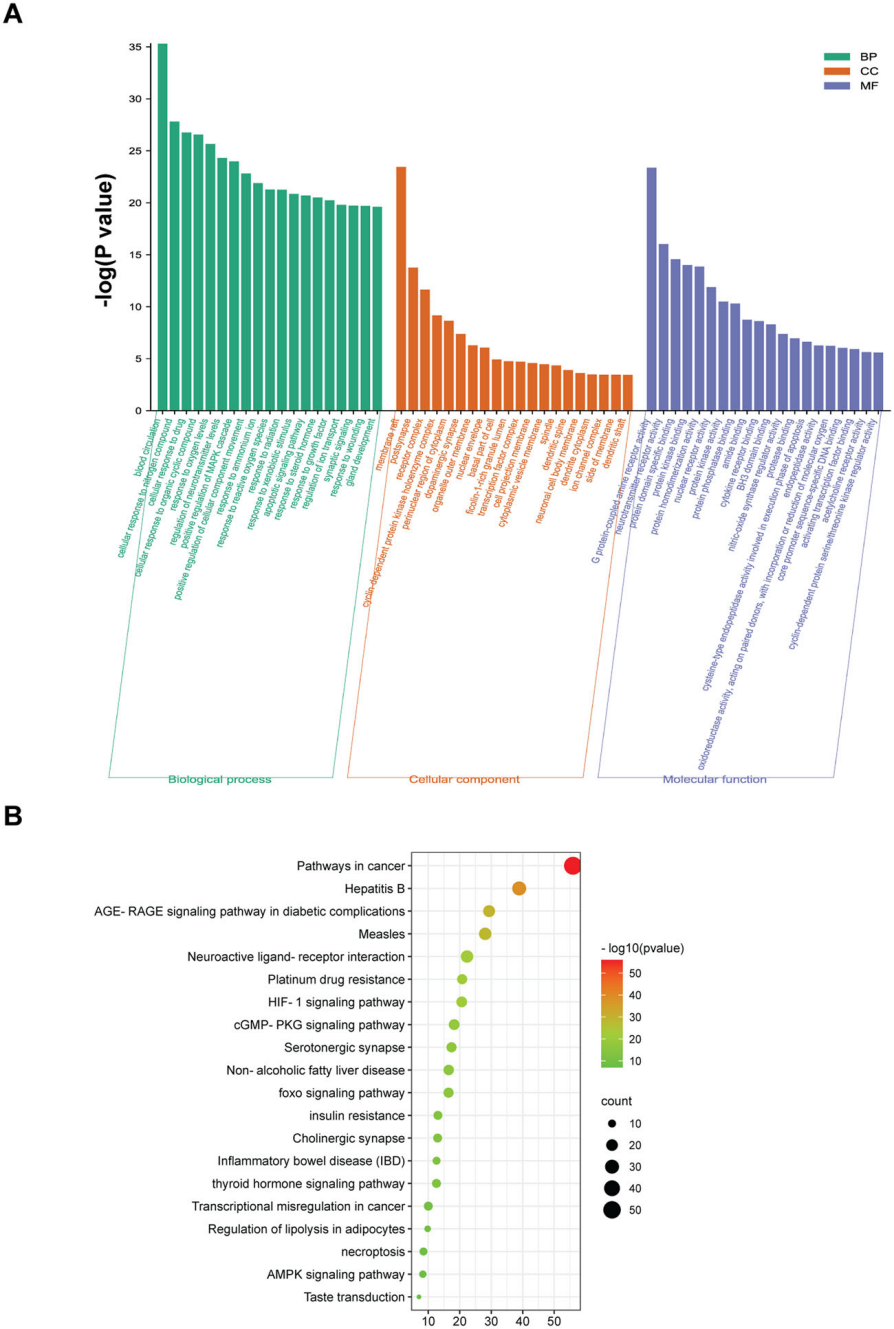
Results of GO enrichment and KEGG pathway analyses of Dan-Shen Decoction targets (A. Molecular function, Biological process, Cellular component; B. KEGG pathway).

The enrichment analyses of molecular function, biological processes, cellular components, and KEGG pathways were performed on the 122 possible targets. The results involved processes such as ‘protein kinase activation’, ‘blood circulation’, ‘growth factor regulation’ and ‘apoptosis’. In the KEGG pathway analyses, the results demonstrated that the ‘cGMP-PKG signalling pathway’, ‘AGE-RAGE signalling pathway’, ‘AMPK signalling pathway’, ‘FoxO signalling pathway’ and ‘HIF-1 signalling pathway’ were the key pathways (Figure 5).

### Molecular docking

**Figure 6.**
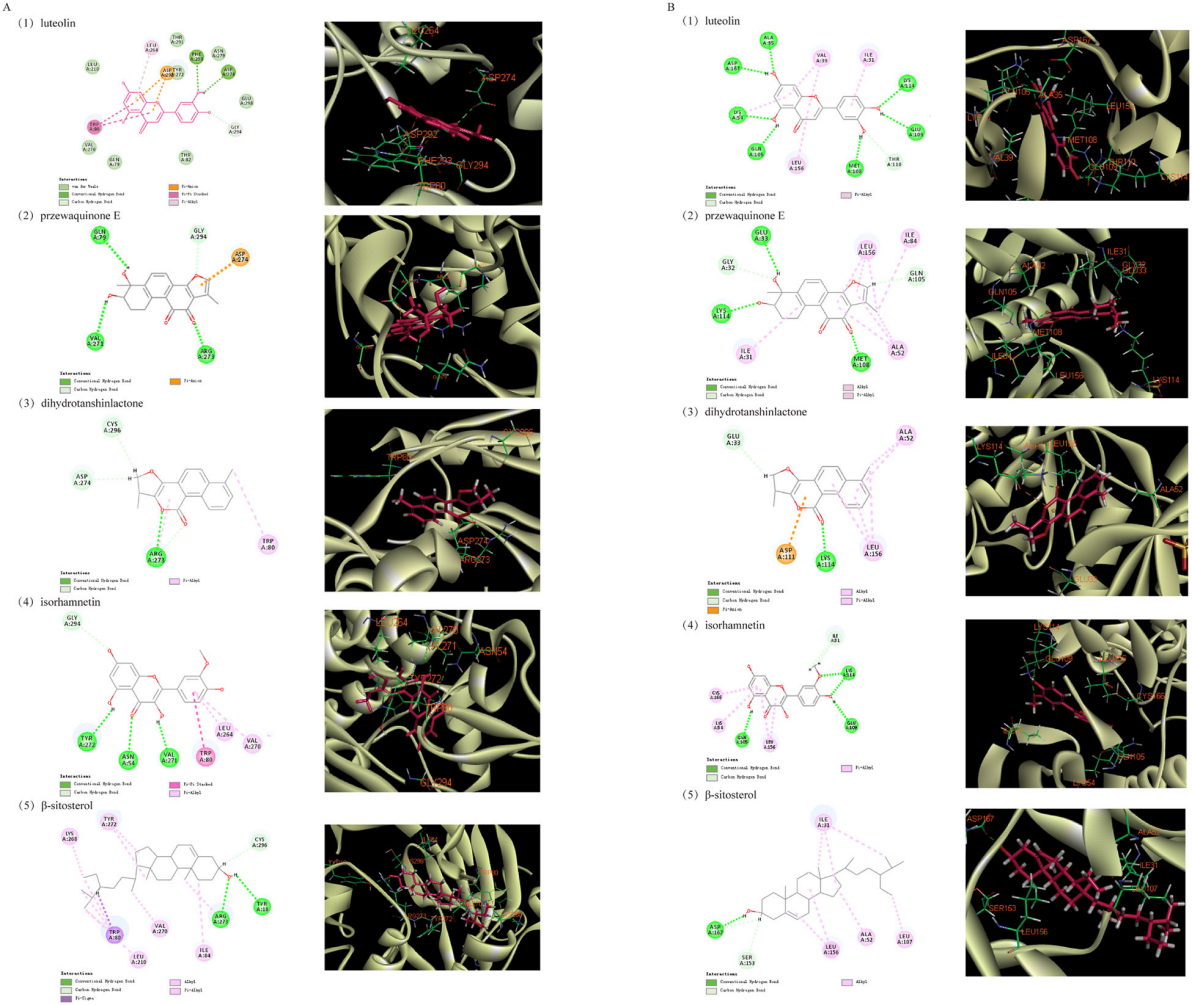
Docking diagram of main active compounds and potential targets (A. Molecular docking with AKT1, B. Molecular docking with MAPK1).

Molecular docking was performed to investigate potential binding modes and the reliability of the interactions between the active compounds and potential targets. Luteolin and isorhamnetin likely interacted strongly with the identified key targets, as suggested by -CDOCKER energies of 35.4522, 36.2697, 36.0682 and 37.0869 kcal/mol (Table 4). In terms of the interaction point, AKT1 mainly interacted with TRP80, LEU264, ARG273, ASP274 and GLY294 amino acid residues in the active site, forming multiple hydrogen bonds and pi-alkyl hydrophobic interactions. The interaction with MAPK1 includes conventional hydrogen bonds with GLU109, LYS114 and ASP167, the Pi-alkyl interaction with ILE31, ALA52 and LEU156, and carbon–hydrogen bonds (Figure 6).

**Table 4.** Interaction binding energy of the main active components of Dan-Shen Decoction with potential targets.

Active components	Targets	-CDOCKER_INTERACTION_ENERGY (kcal/mole)
Luteolin	AKT1	35.4522
	MAPK1	36.2697
Przewaquinone E	AKT1	20.3718
	MAPK1	16.3975
Dihydrotanshinlactone	AKT1	−7.01755
	MAPK1	−8.98489
Isorhamnetin	AKT1	36.0682
	MAPK1	37.0869
β-Sitosterol	AKT1	−18.4643
	MAPK1	−26.258

### Experimental verification

#### Induction of hypoxic H9c2 cells by CoCl_2_

**Figure 7.**
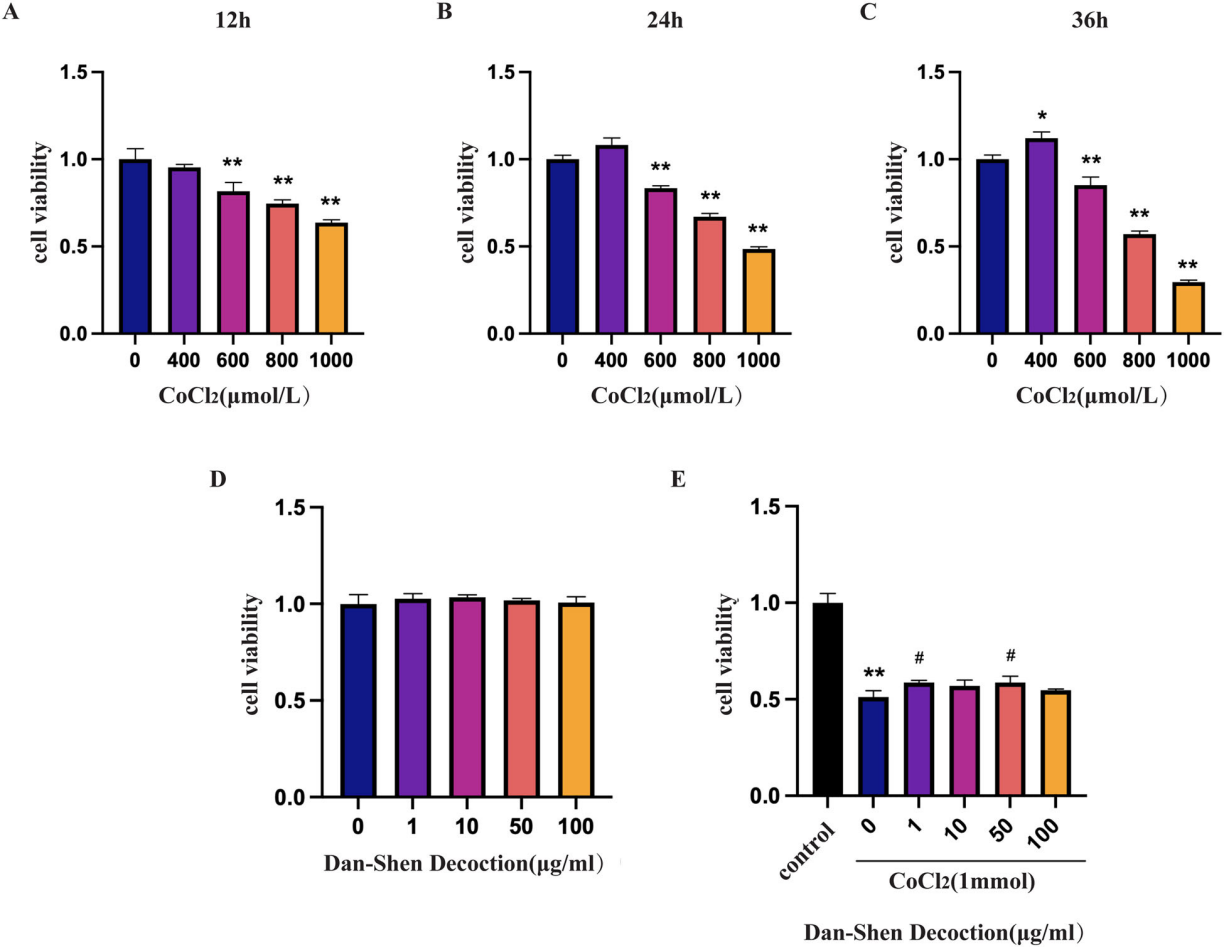
Hypoxia induced by CoCl_2_ in H9c2 cardiomyocytes. A–C. Cell viability of H9c2 cells stimulated with different concentrations of CoCl_2_ (400, 600, 800 and 1000 µmol/L) for 12, 24 and 36 h. *p < 0.05, **p < 0.01 compared with the 0 µmol/L CoCl_2_ group. *n* = 3. D. Cell viability of H9c2 cells induced with various concentrations of Dan-Shen Decoction (1, 10, 50 and 100 µg/mL) for 24 h. E. Cell viability of H9c2 cells pretreated with various concentrations of Dan-Shen Decoction (1, 10, 50 and 100 µg/mL) for 2 h before induction with 1000 µmol/L CoCl_2_ for 24 h. **p < 0.01 compared with the control group. #*p* < 0.05, compared with the CoCl_2_-treated and untreated Dan-Shen Decoction groups (*n* = 3).

Cobalt chloride (CoCl_2_) is a substance that induces hypoxia which is a primary cause of several cardiovascular illnesses, such as IHD, MI and angina pectoris (Chen et al. 2018). The H9c2 cell line was incubated with different concentrations of CoCl_2_ at different times to generate CoCl_2_-induced hypoxic H9c2 cells. The effect of CoCl_2_-induced hypoxia on cell viability was detected by the CCK8 assay. The results showed that the cell viability was reduced by 50% following treatment with 1000 μmol/L CoCl_2_ for 24 h (Figure 7(B)).

We next explored the optimal concentrations of Dan-Shen Decoction to alleviate hypoxic injury. Dan-Shen Decoction was incubated with H9c2 cells at various concentrations. The results showed that concentrations of 1–100 μg/mL Dan-Shen Decoction had no effect on H9c2 cell viability, whereas concentrations of 1 μg/mL and 50 μg/mL had better protective effects on H9c2 cells from hypoxic injury caused by CoCl_2_ (Figure 7(D,E)). Considering the cytotoxicity of the lysis reagent DMSO, the concentration of 1 μg/mL Dan-Shen Decoction was selected for further analyses.

#### Effect of Dan-Shen Decoction on the PI3K/AKT and MAPK signalling pathways in CoCl_2_-induced hypoxic H9c2 cells

**Figure 8.**
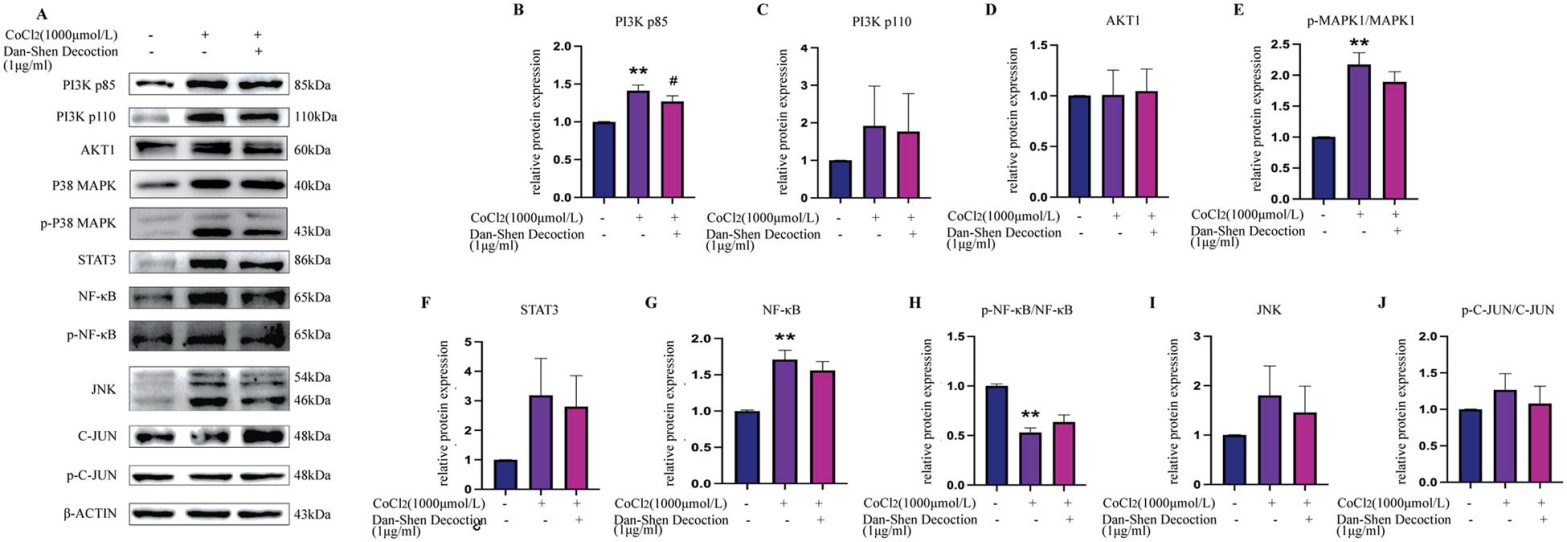
Western blot analyses showed the effect of Dan-Shen Decoction (1 μg/mL) on the expression levels of PI3K/AKT and MAPK signalling pathway-related proteins in control and CoCl_2_-induced hypoxic H9c2 cells. The results are shown as the mean ± SEM. **p < 0.01 compared with the control group, #*p* < 0.05, compared with the CoCl_2_-treated group (*n* = 3).

According to network pharmacology findings, AKT1 and MAPK1, which were the top-ranked proteins in the PPI network, might play critical roles in the therapeutic effects of Dan-Shen Decoction against IHD. Dan-Shen Decoction inhibited the expression of PI3K p110, PI3K p85, STAT3, NF-κB and JNK, decreased the ratio of p-c-JUN/c-JUN and p-MAPK1/MAPK1, and increased the ratio of p-NF-κB/NF-κB (Figure 8).

## Discussion

Dan-Shen Decoction is an herbal compound with various pharmacological effects on the cardiovascular system. Dan-Shen Decoction has been shown to reduce myocardial ischemic burden in patients with unstable angina pectoris (Jiang et al. 2009). Preclinical studies have demonstrated that Dan-Shen Decoction can significantly improve myocardial ischemia induced by isoproterenol or coronary artery ligation (Hu et al. 2012; Wang et al. 2017; Ren et al. 2021). Considering the potential genetic interactions, we aimed to investigate the anti-IHD effects of Dan-Shen Decoction through network pharmacological analyses.

We first identified 122 potential targets through data collection, and luteolin, przewaquinone E, dihydrotanshinlactone, isorhamnetin and β-sitosterol were active components. A study had found that isorhamnetin intake was negatively correlated with the risk of coronary heart disease (Milanlouei et al. 2020), indicating the importance of isorhamnetin in the treatment of cardiovascular diseases. Moreover, PTGS2, SCN5A, ADRB2, OPRM1 and CHRM1 were connected to more active components of Dan-Shen Decoction, which may be the key targets in the treatment of IHD.

The PPI network showed the potential interaction between the 122 targets, and then we selected 15 hub genes from this network based on the degree value. Among them, AKT1 is a serine/threonine protein kinase that is involved in various biological processes such as cell proliferation, metabolism, insulin signalling and angiogenesis (Chen et al. 2017). Recent studies have found that AKT is involved in regulating the biological process of cardiomyocyte apoptosis in the IHD model (Han et al. 2018; Huangfu et al. 2020). The JAK2/STAT3 signalling pathway is thought to be an important anti-IHD pathway because it can regulate myocardial apoptosis and reduce mitochondrial oxidative stress injury (Chen et al. 2020; Lan et al. 2019). Furthermore, the JNK signalling pathway is a critical cellular transduction channel for cardiomyocyte death and plays an important role in the development of cardiac ischemia–reperfusion. Studies have found that salvianolic acid A may increase the expression of thioredoxin (Trx) and inhibit the activation of JNK to reduce apoptosis and inflammation after MI (Zhou et al. 2020).

Recent studies have found that salvianolic acid A can improve endothelial-mesenchymal transition in pulmonary vascular remodelling, inhibit inflammation and oxidative stress, and attenuate vascular remodelling in a pulmonary hypertension model by activating the Nrf2/HO-1 signalling pathway (Chen et al. 2016, 2017). The results of GO enrichment and KEGG pathway analyses also indicated that Dan-Shen Decoction may treat IHD by improving the tension of vascular smooth muscle, inhibiting the inflammatory response and relieving complications.

The results of molecular docking revealed that the three main active components of Dan-Shen Decoction had potent binding effects on two key targets. Among the active components, luteolin and isorhamnetin were flavonoids with more hydrogen bond donors and acceptors, which made them conducive to the formation of hydrogen bonds with the targets and thus more stable binding. Therefore, Dan-Shen Decoction interacted with AKT1 and MAPK1 to regulate multiple targets and signalling pathways and further affected the development of IHD.

To verify the effects of Dan-Shen Decoction *in vitro*, this study established a CoCl_2_-induced hypoxic H9c2 cell model. The expression of PI3K and its downstream proteins AKT1 and NF-κB, as well as MAPK1 and its downstream proteins JNK, STAT3 and c-JUN, was assessed (Chen 2011; Chen et al. 2013). The results showed that Dan-Shen Decoction had no significant effect on the expression of AKT, but it increased the phosphorylation level of its downstream protein NF-κB and inhibited the expression of PI3K p110 and PI3K p85. In addition, Dan-Shen Decoction inhibited the expression of STAT3, NF-κB and JNK, and decreased the phosphorylation levels of c-JUN and MAPK1. The PI3K/Akt/mTOR pathway is a key pathway involved in cell proliferation, differentiation, metabolism, cytoskeleton reorganization and apoptosis (Chen et al. 2017). JNK is a member of the MAPK family, which regulates several cellular functions, including proliferation, differentiation and apoptosis, and can be mediated by ROS to trigger inflammatory responses (Chen et al. 2018). The JAK2/STAT3 pathway, which is the MAPK downstream signalling pathway, protects the ischemic myocardium and alleviates oxidative stress by promoting angiogenesis (Barry et al. 2009; Sui et al. 2019). As previously established, many of the potential targets of Dan-Shen Decoction against IHD are enriched in oxidative stress-related biological processes. Similar results were also obtained in many previous biological experiments (Yan et al. 2012).

Most pharmacology experiments now focus on cellular and animal experiments, which have the advantages of ease of operation and statistics but suffer from the limitation of species differences. Network pharmacology, on the other hand, can supplement traditional pharmacology experiments. The signalling pathways confirmed by basic experiments can also be theoretically justified at the network pharmacology level. The results of this paper provide a good theoretical basis for the study of anti-IHD by providing specific signalling pathways and related target proteins. Additionally, the mechanism of Dan-Shen Decoction against IHD was further examined using molecular docking and preliminary investigations.

However, this study still has some limitations. The key targets and potential pathways obtained from the above analyses should be further explored through biological experiments. Moreover, we only docked AKT1 and MAPK1 with active components of Dan-Shen Decoction in the molecular docking. In a follow-up study, we should not only conduct experiments *in vivo* and *in vitro* to further elucidate the mechanism of Dan-Shen Decoction but also analyze the mechanism with other key targets based on the results of further biological experiments.

## Conclusions

This work demonstrates that several targets, including AKT1, MAPK1, STAT3 and c-JUN, as well as several signalling pathways, including cGMP-PKG, AGE-RAGE, HIF-1, PI3K/AKT, JAK/STAT3 and JNK, are involved in the mechanism of Dan-Shen Decoction against IHD. Although further studies are necessary to elucidate the precise mechanism, this study provides a comprehensive and systematic overview of possible targets and signalling pathways associated with the activities of Dan-Shen Decoction against IHD, providing the theoretical foundation and experimental data for future pharmacological research.

## Consent form

All named authors have agreed to the publication of the work.

## Data Availability

The datasets we generated during the analysis are not publicly available because the analysis process is the core of the results and cannot be made public. Requests to access the datasets of included studies can be met by checking in databases.
